# Improving Diabetes Self-management by Providing Continuous Positive Airway Pressure Treatment to Patients With Obstructive Sleep Apnea and Type 2 Diabetes: Qualitative Exploratory Interview Study

**DOI:** 10.2196/27062

**Published:** 2021-07-20

**Authors:** Ditte Hjorth Laursen, Gitte Rom, Anne Margareta Banghøj, Lise Tarnow, Lone Schou

**Affiliations:** 1 Institute of Nursing University College Copenhagen Copenhagen Denmark; 2 Department of Endocrinology and Nephrology Hillerød Hospital Hillerød Denmark; 3 Steno Diabetes Center Sjælland Holbæk Denmark

**Keywords:** diabetes, diabetes self-management, obstructive sleep apnea, continued positive airway pressure, sleep patterns, sleepiness in daily life, sleep apnea, elderly, sleep

## Abstract

**Background:**

There is a high prevalence of unexplained and unexplored obstructive sleep apnea (OSA) among patients with type 2 diabetes. The daytime symptoms of OSA include severe fatigue, cognitive problems, a decreased quality of life, and the reduced motivation to perform self-care. These symptoms impair the management of both diabetes and daily life. OSA may therefore have negative implications for diabetes self-management. Continuous positive airway pressure (CPAP) therapy is used to treat OSA. This treatment improves sleep quality, insulin resistance, and glycemic control. Although the benefits of using CPAP as a treatment for OSA are clear, the noncompliance rate is high, and the evidence for the perceived effect that CPAP treatment has on patients with type 2 diabetes and OSA is poor.

**Objective:**

The purpose of this study was to analyze the impacts that comorbid diabetes and OSA have on the daily lives of older adults and to investigate the perceived effect that CPAP treatment for OSA has on patients’ diabetes self-management.

**Methods:**

A qualitative follow-up study that involved in-depth, semistructured dyad interviews with couples before and after CPAP treatment (N=22) was conducted. Patients were recruited from the Hilleroed Hospital in Denmark and were all diagnosed with type 2 diabetes, aged >18 years, and had an apnea-hypopnea index of ≥15. All interviews were coded and analyzed via thematic analysis.

**Results:**

The results showed that patients and their partners did not consider OSA to be a serious disorder, as they believed that OSA symptoms were similar to those of the process of aging. Patients experienced poor nocturnal sleep, took frequent daytime naps, exhibited reduced cognitive function, and had low levels of physical activity and a high-calorie diet. These factors negatively influenced their diabetes self-management. Despite the immediate benefit of CPAP treatment, most patients (11/12, 92%) faced technical challenges when using the CPAP device. Only the patients with severe OSA symptoms that affected their daily lives overcame the challenges of using the CPAP device and thereby improved their diabetes self-management. Patients with less severe symptoms rated CPAP-related challenges as more burdensome than their symptoms.

**Conclusions:**

If used correctly, CPAP has the potential to significantly improve OSA, resulting in better sleep quality; improved physical activity; improved diet; and, in the end, better diabetes self-management. However, there are many barriers to undergoing CPAP treatment, and only few patients manage to overcome these barriers and comply with correct treatment.

## Introduction

Type 2 diabetes is one of the most prevalent chronic diseases worldwide and is a lifelong condition with many complications [1,2] that affect the physical, psychological, and social aspects of everyday life [3]. Effective self-management by patients is an important part of diabetes care [4], but poor sleep reduces their motivation to perform self-care and thus impairs their management of both diabetes and daily life [5-7].

A high prevalence of obstructive sleep apnea (OSA) has been documented among patients with diabetes; prevalence estimates range from 23% to 60% [5,8]. This range is much higher than the estimated prevalence rates of OSA in the general population, which are 6% in men and 4% in women [9]. Patients with OSA are often not aware of the nocturnal apneic events that they experience and the fact that daytime symptoms reflect an underlying disorder; thus, they do not seek help [9]. OSA is of great concern for people with diabetes [10]. Further, OSA can result in many complications that affect diabetes self-management. Some of these complications include impaired short-term memory, dietary challenges, and a lack of energy for performing physical activities and taking medicine [11]. Moreover, both OSA and diabetes substantially increase the risk of cardiovascular diseases [12].

OSA is characterized by recurrent episodes of apnea and hypopnea during sleep. It has varying levels of severity, which range from a few to several hundred apneic events per night [13]. Daytime symptoms vary from none to severe fatigue, cognitive problems, a decreased quality of life, and the reduced motivation to perform self-care [5-7]. Medication management, which is critical to diabetes self-management, can also be influenced by daytime sleepiness. The prevalence of OSA increases with age [14], and OSA increases the risk of mortality [13]. A large gap exists in literature related to the potential negative impact that comorbid OSA and diabetes have on daily life. Continuous positive airway pressure (CPAP) therapy is used to treat OSA. In CPAP therapy, a CPAP device is used to deliver air to a mask that patients wear over their nose and mouth and to help patients breathe consistently. Using CPAP as a treatment for OSA can improve sleep quality, mood, and functional status [6]. Furthermore, CPAP reduces the rate of nocturnal urination, cardiovascular disease risk, and the incidence of daytime sleep problems and car accidents among patients with OSA [6]. In addition, adequate CPAP therapy may improve insulin resistance and glycemic control in the long term [7,14]. However, although there are many benefits to CPAP treatment, in some studies, noncompliance rates reach up to 56%, and many patients stop using the CPAP machine during the first month [15]. There is poor evidence on the perceived effect of CPAP treatment and whether CPAP could be related to an improvement in diabetes management among patients with OSA.

The purpose of this study was to analyze the impacts that comorbid diabetes and OSA have on the daily lives of older adults and to investigate the perceived effect that CPAP treatment for OSA has on patients’ diabetes self-management.

## Methods

### Study Design

This was a qualitative follow-up study in which dyad interviews with couples were conducted before and after CPAP treatment. Dyadic interviews draw on the interdependence of two informants as a source of data. Patients with diabetes and OSA and their partners were interviewed simultaneously via joint interviews before and after the intervention [16,17]. Conducting joint dyadic interviews prompts couples to inspire, contrast with, or support each other when exploring their situation [16] because patients often do not even register that they have sleep apnea. This qualitative study was conducted from 2016 to 2017.

### Participants

Participants were recruited from Nordsjællands Hospital, wherein they were a part of a randomized controlled trial—the DiaBOSA (Diabetes and Obstructive Sleep Apnoea) trial (ClinicalTrials.gov identifier: NCT02482584) [18].

The DiaBOSA trial analyzed different disease-related aspects of patients with type 2 diabetes and newly diagnosed OSA. Our qualitative study was a substudy of the DiaBOSA study, which used the same population.

The patient characteristics for this study were a diagnosis of type 2 diabetes; an age of >18 years; and OSA, which was defined as having an apnea-hypopnea index (AHI) ≥15 (measured using ResMed's ApneaLink Air). An AHI of >15 indicates moderate OSA, and an AHI of >30 indicates severe OSA, according to the American Academy of Sleep Medicine guidelines [19]. All participants thus had OSA, even though they were not aware of the diagnosis prior to this study.

During patients’ enrollment in the DiaBOSA trial (N=70), the physician informed the participants about the qualitative study. The authors (DHL and GR) invited the participants, seeking to enroll as many as possibly while also seeking diversity among participants in terms of sex and age. More men than women were enrolled in the DiaBOSA trial. This reflected the proportions of men and women with OSA in general. These proportions were also represented in our qualitative study.

### Data Collection and Analysis

Participants were included until the interviews revealed no new information and the criteria for information power were met. The criteria included a high quality for all interviews and sufficient data for carrying out the analytical strategy [20]. The semistructured question guide covered sleep-related issues that occurred during daily living, the impact that OSA had on participants’ and partners’ lives, an exploration of patients’ sleeping schedules with a special focus on nightly activities, the limitations of daily living resulting from both OSA and diabetes, and the correlation between OSA and diabetes. The questions also broadly focused on all of the concerns surrounding CPAP treatment. In the data analysis, the perspective of the patients was our primary focus; the partners’ perspectives were used to support the statements of the patients.

All interviews took place in patients’ homes, were conducted by 1 or 2 authors, and lasted approximately 60 minutes (range 35-80 minutes). Interviews were recorded, transcribed, and coded using Nvivo version 11 (QSR International).

For the thematic analysis [21], all interviews were read in their entirety and subjected to a systematic coding process. By using Nvivo version 11, a sample of the data was coded individually by the first or second author, after which a coding manual was developed. All interviews were coded by using the manual, which was refined throughout the coding process until all data were organized into meaningful codes. During analyses, the authors discussed any coding uncertainties, and additional codes were developed. Afterward, detailed coded text segments were reviewed and condensed into potential themes [21], and content within themes was reviewed to ensure that they were internally consistent and mutually exclusive.

### Ethical Approval

This study was approved by the Danish Data Protection Agency (journal number: 2012-58-0004). All participants provided written consent after being informed about anonymity and their ability to withdraw from the interview at any time without consequences for future care (in accordance with the Helsinki Declaration).

## Results

### Participants’ Characteristics

The recruited patients—9 men and 3 women—were aged 56 to 75 years; their partners were aged 59 to 75 years. AHIs ranged from 16 to 64; 7 patients had moderate OSA with an AHI of 15 to 29, and 5 had severe OSA with an AHI of >30. The average number of years since type 2 diabetes mellitus diagnosis was 17 years (range 1-44 years), and all patients had elevated hemoglobin A_1c_ levels (range 54-85 mmol/mol; Table 1).

**Table 1:** Patient characteristics (N=12).

Characteristics	Values
Male, n	9
Patient age (years), mean (range)	67 (56-75)
Partner age (years), mean (range)	66 (59-75)
Duration of diabetes (years), mean (range)	17 (1-44)
BMI (kg/m²), mean (range)	33.9 (28.6-39.1)
HbA_1c_^a^ level (mmol/mol), mean (range)	67 (54-85)
Patients with retinopathy, n	2
Patients experiencing elevated urinary albumin excretion^b^, n	4
Vibrations perception threshold (V), mean (range)	25 (10-50)
Patients with cardiovascular disease^c^, n	4
Systolic blood pressure (mmHg), mean (range)	142 (114-174)
Diastolic blood pressure (mmHg), mean (range)	79 (70-91)
Apnea-hypopnea index (number of events per hour), mean (range)	31 (16-64)

^a^HbA_1c_: hemoglobin A_1c_.

^b^A urinary albumin excretion level of above 30 mg/g (albuminuria).

^c^Cardiovascular diseases include myocardial infarction, stroke, and coronary artery bypass graft disease.

Semistructured interviews were conducted with 22 ethnic Danes—12 patients and 10 partners—before they started CPAP treatment. A total of 16 participants (representing both patients and partners) participated in follow-up interviews 3 months after CPAP treatment. Further, 1 patient died during the follow-up period, and 2 patients did not want to participate in the follow-up interviews.

There were large differences in how the participants experienced OSA. Of the 12 patients, 6 had been affected by symptoms of OSA in their everyday lives, while 6 had never anticipated that they would be diagnosed with OSA. Depending on how they experienced their sleep problems, there were differences in how they approached CPAP treatment. These differences had an impact on the structure of the themes for the thematic analysis. A total of 4 overall themes were identified; they are presented in the following sections: *Experiencing Sleep Apnea in Daily Living*, *Changing Sleep Patterns*, *Technical Difficulties in Using the CPAP Device*, and *Part 2: Implications for Diabetes Self-management*. The following results have thus been divided into 2 parts. Part 1 explains the impact that OSA has on daily living and the difficulties in using the CPAP device. Part 2 describes how CPAP treatment has the potential to improve participants’ diabetes self-management.

All 9 follow-up patients were treated with CPAP. The time spent using the CPAP machine varied from 3 days to 3 months.

### Part 1

#### Experiencing Sleep Apnea in Daily Living

There was a clear lack of knowledge about OSA among patients with diabetes. When being diagnosed with OSA, all participants expressed surprise about their diagnosis. One patient said:

I did not think this sleep apnea was serious, but now I can see how serious it is and how many comorbidities that follow.

Patient #1

Both patients and partners frequently commented that they had never anticipated an OSA diagnosis. One patient said:

I had no idea that I had sleep apnea. I have never, ever thought about it.

Patient #5

Some participants thought that being tired was simply part of becoming older. One patient stated:

I have simply interpreted it as old age beginning to present itself.

Patient #4

Although they were told that they stopped breathing during the night, the patients had never taken it seriously or considered it to be a symptom of a disease.

A characteristic feature of OSA is constant sleepiness. Although some patients did not recognize a pattern of persistent sleepiness, it was prominently featured in the experiences of others. This was described by a female patient, as follows:

I’m tired when I get up and I’m tired when I go to bed, right, and I don’t really do anything, because I’m a senior citizen.

Patient #6

Many patients explained that when they are awake at night, they spend their time by using the computer while eating unhealthy snacks.

#### Changing Sleep Patterns

One of the major challenges of sleep apnea is a lack of consistent sleep; patients sleep in short intervals. One patient said:

If I sleep three hours straight, it’s a lot. But it's usually two hours.

Patient #6

Patients did not feel rested when they woke up in the morning after many short intervals of sleep during the night. This often resulted in napping during the day, and up to 4 naps were not uncommon among the study participants. The common belief among patients and partners was that napping was a natural part of becoming older. One partner stated:

When you are 67 years old, you have the right to take a nap, right?

Partner #7

None of the patients viewed napping as a burden. Some of the partners did express concern about their nightly situations and explained what they did to make the patient breathe again. A partner said:

Well, it is not so fun to know that he is lying there not breathing…what if he does not wake up again, right?

Partner #5

The patients who were motivated to continue CPAP treatment did not have more than 1 to 2 hours of continuous sleep for a long period before starting treatment. This had had a major impact on their quality of life. Thus, they were motivated to use the CPAP device. They used the CPAP device every night for 4 to 6 hours and experienced immediate beneficial effects. A patient stated:

Then in the months I've had CPAP I've been sleeping 5-6 hours every night. The first night I slept for seven hours, uninterrupted.

Patient #5

Another benefit that patients experienced was waking up less frequently, and if they did wake up, it was only for a short period. One patient said that before CPAP treatment, he was awake for 2 to 3 hours every night. However, after CPAP treatment, he was only awake for half an hour. Several patients indicated that after CPAP treatment, the quality of their sleep changed; they slept better, quieter, and deeper at night.

#### Technical Difficulties in Using the CPAP Device

Despite the immediate benefit of CPAP treatment, most of the patients experienced technical challenges when using the CPAP mask and machine. The patients who were motivated to undergo CPAP treatment quickly became accustomed to using the CPAP device. One of the typical difficulties that patients experienced was finding the mask that best fit them. Patients who experienced persistent technical challenges typically used the CPAP device for 1 to 2 hours per night if they could make it work at all. Patient #12 had to try 4 different masks before he found one that worked. A small proportion of patients (2/12, 17%) opted to stop CPAP therapy because they could not make the device work properly. Furthermore, some of the patients only tried using the mask and machine a few times before stopping CPAP therapy. For example, one patient said:

Well, I had it one night and I took it off. I could not rest at all.

Patient #7

The main technical challenge was that the mask did not fit the shape of patients’ faces. The CPAP mask did not fit tightly; therefore, air blew into patients’ eyes, which made them very uncomfortable. Patients were also concerned about the hose, which can be too short or too long; they were worried that the hose would crack. Some of the partners felt annoyed by the sound of air and noise from the machine. Further, some patients found that they could not draw air and breathe freely and thus felt like they were being suffocated. One patient stated:

Yes, I did [panic], because I wasn't fully awake, and I couldn't get the mask off. It was awful.

Patient #7

When using the CPAP device, one must breathe with their mouth shut and exhale with resistance. This was a challenge for most patients, but some became accustomed to it. One patient said:

I feel I need to adjust my breathing to the machine, but I shouldn't, it does it for me. But I’m getting used to it and it's getting better and better. And then I learned how to slow down the blowing from the machine which helped.

Patient #5

### Part 2: Implications for Diabetes Self-management

All participants reported a decline in their memory over the previous few years. This decline in short-term memory was of great concern to all patients and their partners. As several couples were very worried about this, several patients underwent testing for dementia; all tests were negative.

As shown in Table 1, all patients were receiving treatment for diabetes. Nearly all patients had developed daily routines for taking their medications on time, such as placing small notes on a table or using an alarm. Unfortunately, the notes were often misplaced, and patients often forgot to take their medicine. Additionally, two patients reported struggling with medication management due to sleepiness. One patient was often asleep when she was supposed to take her medication and struggled to manage her blood glucose levels. All patients knew that they should have a healthier diet. However, in their daily lives, they ate what they wanted and did not focus on their diabetes. Other couples explained that they often did not plan dinner in advance, which frequently resulted in eating fast food.

Participants reported consuming large amounts of candy and cake. Despite knowing that they should not eat sweets, many could not help themselves. For example, one partner said:

He feels like an addict if there is a cake.

Partner #2

Even though many participants reported that they ate many sweets, no one thought that this was related to OSA.

Several participants described being physically active during their youth. However, at the time of the baseline interviews, more than half of participating patients (9/12, 75%) did not exercise at all, and those who were still active had reduced their exercise intensity substantially. After initiating CPAP treatment, changes were observed in factors related to patients’ diabetes self-management. First, several of the patients felt well rested and healthier in the morning, and this feeling had a direct influence on their memory. They missed fewer appointments with their health care provider and could more easily recall when to take the right medicine. Some of the patients also described having the energy to eat healthier, exercise, and manage work tasks that they were not been able to perform for several years. One of the patients lost 10 kg during the 3-month test phase due to experiencing better sleep, which was the result of no longer eating during the night and being less hungry for snacks during the day. Several participants also explained how they started exercising again due to having more energy and being less tired. A combination of regular medicine intake, a well-regulated diet, and an increase in activity levels also improved several of the participants’ glucose levels. Due to improved sleep, patients needed fewer naps during the daytime, which improved their social relationships and had a positive impact on their partners.

## Discussion

### Principal Findings

Patients with OSA and diabetes and their partners did not consider OSA to be a serious disorder that affected daily living. Despite some patients’ experiences with excessive sleepiness during the day, they interpreted sleepiness and napping as natural components of aging and organized their daily activities accordingly. Since the symptoms of OSA were not familiar to participants, they did not associate these symptoms with a disease. The main outcome from the baseline interviews was that these patients performed limited amounts of daily physical activity, experienced challenges with short-term memory, and had a high intake of sweets. These factors had implications for their diabetes self-management. After using the CPAP device for 3 months, wide variations appeared among the patients. Many patients experienced technical difficulties with using the CPAP machine, which made them stop the treatment. This was often related to the fact that the mask did not fit their faces. Only the patients who were motivated to change their sleeping patterns overcame the technical difficulties. The patients who adhered to CPAP treatment after 3 months lost weight, improved their level of physical activity, improved their food intake, and had more energy in their lives. These improvements all correlated with their diabetes self-management.

### Disease Management as a Daily Routine

The results show that a chronic condition can become a natural part of everyday routines. Study participants did not make conscious changes to their daily living due to a disease. Rather, their symptoms were integrated into their routines. This has been described in other studies of chronic illness [22,23]. Previous research has shown that patients correlate their feelings of being sleepy with laziness and do not associate such feelings with a medical diagnosis. This may explain why our participants adjusted their daily lives around their symptoms. Moreover, symptoms of OSA are both nonspecific and similar to those of the natural process of becoming older [24]. This provides ample room for patients’ personal explanations and interpretations.

### Concerned Partners

Very few studies have analyzed partners’ perspectives on OSA, and most findings have indicated that partners are afraid and that many partners monitor patients’ breathing at night [25,26]. Partners have also indicated that although they consider snoring to be annoying, it also provides reassurance that the patient is still breathing. We observed notable differences in partners’ experiences of nighttime disturbances. Some did not mind patients’ snoring and could either turn off a hearing aid or use the snoring as a kind of meditative white noise for falling asleep. Others demonstrated the findings described above and were worried that their partner might not wake up after an apneic episode. A lack of worry could be related to ignorance about OSA and its consequences.

### Changed Cognitive Function

Several participants experienced challenges with short-term memory and developed corresponding strategies to overcome these challenges. Some even took a dementia test. A review found that patients with OSA and excessive daytime sleepiness may have cognitive impairments related to attention, concentration, learning, memory, and executive functions [14]. The most likely reasons behind cognitive declines are sleep problems and hypoxia. Changes in cognition may negatively affect diabetes self-management behaviors, thereby influencing self-care outcomes, planning, and problem solving [27-29]. Specifically, a review showed that poor cognitive function negatively influences diabetes-specific numeracy abilities, insulin adjustment skills, adherence to medications, the frequency of performing self-care activities, the number of missed appointments, the frequency of diabetes monitoring, and the accuracy of reported blood glucose levels [29]. It is therefore important to be aware of changes in cognitive function among patients with comorbid diabetes and OSA.

### Perceptions of OSA Severity Determines the Use of CPAP

Patients who do not have symptoms of fatigue and a tendency to fall asleep during the day but experience many technical challenges in CPAP treatment are less likely to continue treatment. This may be related to the fact that they doubt their OSA diagnosis and the risk of complications, as they have no obvious symptoms [24]. This was also observed in a study by Sawyer et al [30], who found that perceptions and assessments of the risk of OSA, the recognition of symptoms, and expectations of improvement in sleep are different between patients who adhere to CPAP therapy and those who do not. It was also seen in our study that patients who adhered to CPAP treatment were prepared to cope with technical and breathing challenges if they slept well at night and had more energy during the day. Patients who did not adhere to CPAP treatment did not experience the benefit of the treatment. These results are in line with those of other studies that underline the importance of using the correct mask and machine to improve sleep [31,32]. Several studies have shown that patients' experiences and perceptions of symptoms and the assessment of OSA consequences are of great importance for accepting an OSA diagnosis and undergoing CPAP treatment. Therefore, these factors are also important for increasing patients’ motivation to continue treatment and their commitment to the treatment [30,32].

In addition to sleeping better at night, many patients had higher energy levels during the day, resulting in the need for fewer and shorter naps as well as increased desires and energy for performing activities. These findings are supported by several studies that show that patients undergoing CPAP treatment report less snoring, longer coherent sleep, higher energy levels, fewer conflicts with family and others, better memory, and greater activity levels [33,34].

Some partners also experience the changes that patients exhibit, such as being in a better mood during the day as well as being awake for fewer and shorter periods at night. Studies that focus on couples' experiences with CPAP therapy have shown that partners can motivate and support patients both emotionally and practically in their use of a CPAP mask. Patients have shown consideration and do not interfere with their partners at night by using CPAP, thereby motivating patients to adhere to treatment [34,35].

### Technical Challenges in Using the CPAP Device

Patients’ challenges with mask customization often resulted in adverse consequences in the form of eye inflammation and clogged and runny noses [26,33,34,36-38], which may have reduced their motivation to continue using the CPAP device. Some of the patients and partners also felt annoyed by the sound of “air leaking next to the mask” and noise from the machine. Findings from other studies have suggested that these disturbances in sleep, sleep routines, and intimacy are perceived as barriers, and partners may be concerned about patients’ use of the mask and machine [34,35]. Some of the patients in our study had breathing difficulties associated with CPAP treatment and were afraid of being unable to breathe. Studies have shown that patients, especially at the beginning of CPAP treatment, are afraid of wearing the mask and are challenged by the lack of control over their breathing [39,40]. These technical and respiratory challenges present a high risk of discontinuing treatment among patients; CPAP treatment has a tendency to induce feelings of claustrophobia and anxiety, which may affect patients’ adherence to CPAP therapy [30,41,42]. Interventions that focus on biomedical, psychological, and social factors in CPAP treatment have resulted in positive adherence to CPAP treatment [30,43].

### Dietary Improvements After CPAP Treatment

The dietary implications of OSA are highly relevant to patients with diabetes. Patients with OSA prefer more calorically dense food with a higher fat content [44,45] and have verbalized the importance of maintaining their diet. However, they have also stated that they often feel too sleepy to do anything beyond the minimum for what is expected of them [6]. The increase in their intake of high-calorie foods may be related to the effects that interrupted sleep and apnea have on the secretion of the hormones leptin and ghrelin. These effects result in a resistance to leptin and an increased secretion of ghrelin [45]. Individuals with OSA often feel hungry and prefer high-calorie food. Experiencing wakeful periods several times per night increases the likelihood of nocturnal food intake, which negatively affects glucose balance and obesity and, in turn, negatively impacts both OSA and diabetes [46,47]. Obesity is a major risk factor for OSA. The prevalence of OSA is high among patients with obesity and vice versa [48]. Weight loss may result in an improvement in the severity of OSA and, perhaps, even its resolution [48], which would also improve diabetes.

In our study, some patients and partners benefitted from dietary changes and minor weight loss. This can be understood in the context that several of the patients undergoing CPAP treatment slept better at night and had higher levels of energy and activity during the day. In a study by Bakker et al [37], it was pointed out that improvements in OSA symptoms such as snoring, apnea, and a tendency to fall asleep during the day motivate patients during CPAP treatment, while increased energy levels per day motivate patients to change their lifestyles (eg, some patients change their diet and exercise habits).

One of the patients in our study lost 10 kg during the study period, and this had an immediate effect on his glucose levels. Consequently, dietary changes and weight reduction should be particularly emphasized in the treatment of both OSA and diabetes.

### Physical Improvements After CPAP Treatment

With regard to physical functions, OSA has implications for physical activity levels and the experience of subjective vigor [7]. In our study, patients reported a decline in physical activity but did not associate this with sleepiness or OSA. Instead, they described this decline as a result of a lack of motivation, lack of energy, or other chronic diseases. However, patients who were using the CPAP device correctly immediately experienced improvements in their daily levels of activity. In addition to treatment via CPAP, OSA can be improved with physical exercise. Patients with OSA who were involved in a regular, predominantly aerobic exercise program exhibited reduced levels of disease severity and daytime sleepiness as well as increased sleep efficiency and peak oxygen consumption, regardless of weight loss [49,50]. This underscores the potential value of exercise in OSA management and the importance of providing information to patients about the value of physical activity to improve both OSA and diabetes.

### Strengths and Limitations

The primary strength of this study lies in our use of qualitative methods. As there is a very high prevalence of undiagnosed OSA in the population [51], new study designs are needed to understand the correlation between the two diseases. By using a qualitative design, we revealed that most patients experienced OSA symptoms. However, such information would not be captured by a questionnaire. Another strength is that we conducted dyadic interviews with patients and partners. Dyadic interviews can inspire informants to provide more information than they would during two individual interviews; however, there is a risk that some things might be left unsaid to avoid confrontation or offense.

There are some limitations to this study. Patients were recruited from a randomized controlled trial study and via self-selected participation. Therefore, they may differ from comparable patients with OSA and diabetes who chose not to participate. Additionally, only patients with moderate or severe OSA (an AHI of >15) were included; they were the most likely to exhibit daily symptoms and feel restricted by OSA. Further, our findings may not be generalizable to patients with an AHI of 5 to 15, about whom little is known in terms of treatment and how they live with OSA during their daily lives. Finally, the OSA diagnoses were made via polygraphy, which probably underestimated the AHI and subsequently misclassified patients with OSA.

### Conclusion

In this study, we analyzed a group of older adults with comorbid OSA and diabetes and found that their lives were disrupted due to the two diseases and were characterized by poor sleep and frequent naps. Most often, patients are unaware of their OSA and do not seek treatment. The implications of reduced cognitive function, low levels of physical activity, and a high-calorie diet affect diabetes management and result in exacerbated diabetes. If used correctly, CPAP has the potential to significantly improve OSA, resulting in better sleep quality; improved physical activity; improved diet; and, in the end, better diabetes self-management. Nevertheless, there are many barriers to undergoing CPAP treatment, and only few patients manage to overcome these barriers and comply with correct treatment. Patients’ partners play a large role in promoting the correct use of the CPAP device, which can motivate patients to continue with the treatment. Patients who correctly use the CPAP device also exhibit improvements in their diabetes self-management. It is therefore important that CPAP-related barriers are prevented via thorough instruction and assistance from health care professionals.

It is also important that patients and their relatives acquire knowledge about the symptoms of and risk factors for OSA and understand the connection between OSA and diabetes. Patients and their relatives must be aware that changes in sleep patterns, increases in fatigue during the day, and the need for extra naps can be symptoms of OSA. Thus, there is also a great need for increasing OSA awareness among health care professionals, so that they can learn to identify affected individuals and develop skills for providing screening, education, and guidance based on the needs of patients and their partners. As a result, patients can gain an understanding of OSA and skills for managing symptoms in daily life.

## Abbreviations

AHI: apnea-hypopnea index
CPAP: continuous positive airway pressure
DiaBOSA: Diabetes and Obstructive Sleep Apnoea
OSA: obstructive sleep apnea
